# Advantages of Highly Spherical Gold Nanoparticles as Labels for Lateral Flow Immunoassay

**DOI:** 10.3390/s20123608

**Published:** 2020-06-26

**Authors:** Nadezhda A. Byzova, Anatoly V. Zherdev, Boris N. Khlebtsov, Andrey M. Burov, Nikolai G. Khlebtsov, Boris B. Dzantiev

**Affiliations:** 1 A.N. Bach Institute of Biochemistry, Research Center of Biotechnology, Russian Academy of Sciences, Moscow 119071, Russia; nbyzova@mail.ru (N.A.B.); zherdev@inbi.ras.ru (A.V.Z.); 2 Institute of Biochemistry and Physiology of Plants and Microorganisms, Russian Academy of Sciences, Saratov 410049, Russia; burov_a@ibppm.ru (A.M.B.); khlebtsov_n@ibppm.ru (N.G.K.); 3 Saratov State University, Saratov 410012, Russia

**Keywords:** immunochromatography, nanosized labels, nanoparticle–antibody conjugates, assay sensitivity, cardiomarker

## Abstract

The use of lateral flow immunoassays (LFIAs) for rapid on-site testing is restricted by their relatively high limit of detection (LoD). One possible way to decrease the LoD is to optimize nanoparticle properties that are used as labels. We compare two types of Au nanoparticles: usual quasispherical gold nanoparticles (C-GNPs), obtained by the Turkevich–Frens method, and superspherical gold nanoparticles (S-GNPs), obtained by a progressive overgrowth technique. Average diameters were 18.6–47.5 nm for C-GNPs and 20.2–90.4 nm for S-GNPs. Cardiomarker troponin I was considered as the target analyte. Adsorption and covalent conjugation with antibodies were tested for both GNP types. For C-GNPs, the minimal LoD was obtained with 33.7 nm nanoparticles, reaching 12.7 ng/mL for covalent immobilization and 9.9 ng/mL for adsorption. The average diameter of S-GNPs varied from 20.2 to 64.5 nm, which resulted in a decrease in LoD for an LFIA of troponin I from 3.4 to 1.2 ng/mL for covalent immobilization and from 2.9 to 2.0 ng/mL for adsorption. Thus, we obtained an 8-fold decrease in LoD (9.9 to 1.2 ng/mL) by using S-GNPs. This effect can be related to more effective antibody immobilization and improved S-GNP optical properties. The obtained results can improve LFIAs for various practically significant analytes.

## 1. Introduction

Lateral flow immunoassay (LFIA)—also known as immunochromatography—has been suggested as an effective analytical method for point-of-care diagnostics [1,2]. The design of the immunochromatographic test strip with pre-applied reagents ensures the autonomous implementation of all analytical processes. The assay can be initiated by a simple contact of the test strip with the sample and does not require additional manipulations with reagents and devices. A quick immunospecific reaction (5–15 min) leads to the formation of visually detectable stained zones in certain areas of the test strip with nanoparticle-labeled immune complexes [3,4]. However, the fast reaction kinetics and absence of the additional signal amplification step lead to the relatively low sensitivity of LFIA compared to other types of immunoassay.

Various approaches have been considered for increasing LFIA sensitivity, including multistep analysis and specific detection techniques [5,6,7]. However, such improvements result in the loss of the main advantage of LFIA as a simple point-of-care test. A promising approach is to optimize nanoparticles used as labels without significant changes to simple optical (including naked eye) detection. The optimal LFIA labels should meet several quality criteria, including ease of preparation, high optical response, and the saving of antibodies’ affinity during conjugation [8]. Most of the existing colorimetric immunochromatographic systems are based on the use of gold nanoparticles (GNPs). The advantages of GNPs are connected to their high extinction cross-section in the visible spectral range due to surface plasmon resonance, well-known protocols for the synthesis of monodispersed colloids with designed nanoparticle size, a long history of conjugation with antibodies [9,10], and identified properties of the obtained preparations [11,12,13,14].

The most widely used GNP synthesis method is based on sodium citrate’s reduction in gold salts. Turkevich [15] suggested this method. The method was further improved to reduce the heterogeneity of the synthetic products in size and shape [16,17,18,19]. However, the published modifications to the method did not solve this task completely; the described variants either retained the heterogeneity of the obtained GNPs or they proposed a method for producing homogeneous GNPs, limited to a single preparation of a certain diameter. Thus, N.G. Bastus et al. [20] proposed a two-stage synthesis that significantly expands the size range for stable GNPs with respect to the traditional one-stage Turkevich–Frens technique. However, the relative standard deviation (RSD) of the GNPs diameter for these preparations varied in the range of 5–9%. For other known works, improvements in homogeneity are either relatively moderate or accordant to the only preparation of a small diameter. Thus, J. Dong et al. [21] described a technique that decreased the RSD of the GNPs diameter to 8–10% with an aspect ratio of 1.10–1.22 (for two GNP preparations). J. Kimimg et al. [17] modified the Turkevich–Frens method, providing an RSD of the GNP diameter from 13% to 16% for a preparation range of up to 40 nm. F. Shiba [22] described a finely dispersed preparation with an RSD of 7.6%, but this was only the case where the diameter was equal to 14 nm. Schulz F. et al. [23] reached the gain in homogeneity with a decrease in RSD from 8% to 3%, but only for small GNPs with a diameter of 12 nm. H. Xia et al. [24] described an improved synthesis of citrate GNPs (C-GNPs) in the 12–36 nm range characterized by an RSD of 9% or higher. E. Méndez and S. Botasini [25] recently reported C-GNPs obtained with a polydispersity index of not more than 5% for a particular preparation with a diameter of 14 nm. Commercial manufacturers reported GNPs with a very high homogeneity in a wide range of diameters [26,27,28], but the protocols of their syntheses remain the know-how for developers.

Despite the available wide range of GNP sizes, the question of the optimal size for LFIA is still under debate, including the impact of GNPs’ shape on this choice. Basically, the large sizes of GNPs allow a target (controlled) molecule to be labeled with a large number of gold atoms. However, the literature does not give a reason to believe that the differences in detection limits of immunochromatography are determined primarily and exclusively by GNP size. The comparative consideration of GNPs with different diameters in immunochromatography [29,30,31,32,33,34] indicates that the change in LFIA sensitivity with an increase in GNP size is nonmonotonic.

Some experimental results show that 30–40 nm GNPs are optimal [17,35,36] because smaller particles have unacceptably small extinction cross-sections and small surface areas for antibody internalization. On the other hand, we recently demonstrated that increasing the nanoparticle size up to 115 nm reduced the LoD [37]. Moreover, conclusions about the capabilities of a label can be made only by comparing the analytical characteristics achieved for its conjugates with antibodies of different compositions [38,39,40,41,42]. However, changes in the antigen-binding properties of antibodies with variation in their surface density on a nanoparticle are nontrivial and, to date, have not been described by a single, universally recognized model. For example, the curvature of the nanoparticle surface can play an important role in the affinity of the GNP–antibody complex [43].

From this point of view, superspherical GNPs (S-GNPs) can be more suitable labels for LFIA instead of the usual quasispherical nanoparticles obtained with the Turkevich–Frens method. The particles used for LFIA require, at least, high stability of colloidal dispersions, excluding their aggregation and nonspecific binding on the membrane. In this regard, the absence of fluctuations in the particle surface is an important potential advantage of superspherical GNPs. The unified surface properties of superspherical GNPs reduce their nonspecific interactions. Monodispersed colloids of S-GNPs can be obtained using seed-mediated growth in a cetyltrimetylammonium bromide solution [29,44]. The other important advantages of S-GNPs are related to high colloidal stability in a wide range of sizes and stable optical properties that can be finely described by the Mie theory [29]. Despite these advantages, S-GNPs have not been previously tested as labels for LFIA.

Here, we compare the LoD of LFIA strips based on S-GNPs and conventional C-GNP synthesized according to the Turkevich–Frens method. For a grounded comparison, a set of GNPs with different diameters was synthesized and conjugated with antibodies by using simple physical adsorption and covalent binding using a succinimide-thiol crosslinker. As an antigen, troponin I (cardiac isoform) was chosen, which is widely used in medical diagnostics as a biomarker of acute myocardial infarction [45]. Due to the variability of its release into the bloodstream, systems of highly sensitive detection of this compound have been recently developed, both immunoenzymatic (successfully introduced into practice) and immunochromatographic [46,47]. In this regard, an assessment of the possibilities of applying the novel S-GNPs in LFIA, which do not require the complications of the testing methodology, is of great importance.

## 2. Materials and Methods

### 2.1. Reagents and Materials

The following antigens and antibodies were used in this study: native human troponin I (cardiac isoform, cTnI), monoclonal anti-cTnI antibodies, IC4 and IC19 clones (all from Bialexa; Moscow, Russia), and goat anti-mice immunoglobulin antibodies (GAMI; Arista Biologicals; Allentown, PA, USA). Cetyltrimethylammonium bromide (CTAB, 96%; Fluka), cetyltrimethylammonium chloride (CTAC; 25% water solution), L-ascorbic acid (AA, >99.9%), sucrose, sodium borohydride (NaBH_4_, 99%), 3,3′,5,5′-tetramethylbenzidine dihydrochloride (TMB), tris(hydroxymethyl)aminomethane (Tris), Tween 20, Triton X-100, dimethyl sulfoxide (DMSO), sodium citrate, and sodium azide were purchased from Sigma–Aldrich (St. Louis, MO, USA). Tetrachloroauric acid (HAuCl_4_, 99.99%) was procured from Alfa Aesar (Haverhill, MA, USA). Bovine serum albumin (BSA) came from Biowest (Nuaille, France). Thiolated polyethylene glycol (PEG-SH, MW = 5000) and orthopyridyl disulfide polyethylene glycol succinimidyl ester (OPSS-PEG-NHS, MW = 5000) were obtained from Creative PEG Works (Chapel Hill, NC, USA). All auxiliary reagents (e.g., salts, acids, alkalis, organic solvents) were of analytical or chemical purity. All chemicals were used without further purification.

GNP synthesis solutions and their conjugation with antibodies were prepared in deionized water (simplicity system, Millipore; Bedford, MA, USA; specific resistivity at 25 °C was ≥18.2 MΩ cm). To manufacture lateral flow test strips, the following membranes were used: a nitrocellulose (NC) membrane grade CNPC with a pore size of 15 μm attached to a solid support, conjugate release matrix PT-R7, sample membrane GFB-R4, and absorption membrane AP045 (all membranes from Advanced Microdevices; Ambala Cantonment, India). ELISA was performed in transparent 96-well Costar 9018 polystyrene microplates (Corning Costar; Corning, NY, USA).

### 2.2. Synthesis of Gold Nanoparticles Using the Citrate Method

Citrate-capped gold nanoparticles were synthesized via the Frens method [16] with modifications according to our paper [48]. An aqueous solution of HAuCl_4_ was added to deionized water, as indicated in Table 1, and the mixture was brought to a boil. Then, a solution of sodium citrate (see Table 1) was added with stirring. The mixtures were boiled for 25 min, and then cooled and stored at 4–6 °C. The obtained colloids were labeled C-GNPs-1–C-GNPs-5.

### 2.3. Synthesis of Spherical Gold Nanoparticles

The superspherical gold nanoparticles were made by using a protocol described elsewhere [29,33]. In the first step, 1−3 nm gold seeds were prepared by adding of 600 μL of NaBH_4_ (10 mM) to a mixture containing 5 mL of aqueous CTAB (0.2 M) and 5 mL of HAuCl_4_ (1 mM). Then, 10-nm GNPs were prepared by adding 20 mL of 0.5 mM HAuCl_4_ to a mixture containing 20 mL of CTAC (0.1 M), 15 mL of AA (0.1 M), and 0.5 mL of seeds. After 30 min of the reaction, the resulting 10-nm GNPs were centrifuged at least thrice at 20,000 *g* for 60 min. Finally, 10-nm GNPs were resuspended in 10 mL of 0.1 M CTAC. Then, these 10 nm GNPs were overgrown to a designed size. To this end, 0.1 M CTAC, 10 mM AA and the 10-nm GNPs were mixed, as indicated in Table 2, in a 200-mL flask. Further, 2 mM HAuCl_4_ was added by using a syringe pump at the injection rate 10 mL/h. Finally, the S-GNPs were centrifuged at 1000–15,000 *g* and resuspended in water to have an optical density of about 1.5. The obtained colloids were labeled as S-GNPs-1–S-GNPs-5.

### 2.4. Transmission Electron Microscopy (TEM)

The C-GNP preparations were applied to 300-mesh grids (Pelco International; Redding, CA, USA) coated with formvar film. Images were acquired with a CX-100 electron microscope (Jeol; Tokyo, Japan) at an accelerating voltage of 80 kV. Digitized images were analyzed with Image Tool software. TEM images of S-GNPs were recorded with a Libra 120 microscope (Carl Zeiss; Germany) at the Simbioz Center for the Collective Use of Research Equipment in the Field of Physical–Chemical Biology and Nanobiotechnology, IBPPM RAS, Saratov.

### 2.5. Dynamic Light Scattering (DLS) Measurements of GNPs and Their Conjugates

Dynamic light scattering was measured using a Malvern Zetasizer Nano (Malvern, UK). Statistical data processing was performed by Malvern Softwarever 7.11 (Malvern, UK). Diameter determination of particles was performed in the range from 0.3 nm to 10 μm.

### 2.6. Estimation of C-GNPs and S-GNPs Using ICP-MS

The Au concentration in the C-GNP and S-GNP solutions was obtained by inductively coupled plasma mass spectrometry (ICP-MS), as described in our paper [39]. The ICP-MS measurements were carried out with a quadrupole ICP-MS instrument Aurora M90 (Bruker Corp., Billerica, MA, USA) equipped with a MicroMist low-flow nebulizer (equipment of the Shared-Access Equipment Center «Industrial Biotechnology» of Federal Research Center «Fundamentals of Biotechnology», Russian Academy of Sciences, Moscow, Russia). A series of Au standard solutions (0.1−5.0 ppb in 1% HCl (*v/v*) were prepared before each experiment. Scandium was used as the internal standard, eliminating the fluctuations coming from the measuring conditions. All samples were prepared in triplicate. Quantum software (Bruker Corp., v 3.1) was used for data collection and processing. In the calculations of GNP concentrations, the Au density was equal to 19.3 g/cm^3^ and the volume of one particle was 4/3·πr^3^, where r was half the sum of the half-mean major and half-mean minor axis lengths obtained by TEM.

### 2.7. Adsorption Immobilization of Antibodies on GNPs

GNPs were functionalized with anti-cTnI monoclonal antibodies and clone IC4. GNP solutions (pH 9.0) were added to antibody solutions at the proportions indicated in Section 3.2. The mixture was incubated at room temperature for 30 min under stirring, after which an aqueous BSA solution was added to a final concentration of 0.25% (*w/v*). GNPs with immobilized antibodies were separated from unreacted antibodies by centrifugation for 15 min at the accelerations indicated in Table 3. After the supernatant liquid was discarded, the sediment was resuspended in 0.02 M Tris–HCl buffer (pH 7.6) containing 1.0% BSA, 1.0% sucrose, 1.0% Tween 20, and 0.1% sodium azide (all *w/v*). The preparations were stored at 4–6 °C.

### 2.8. Covalent Immobilization of Antibodies on GNPs

Covalent immobilization of the IC4 antibodies on GNPs was carried out according to our previous paper [49]. First, the succinimidyl carboxymethyl ester (OPSS–PEG–NHS) cross-linker reacted overnight with the antibodies at a 10:1 molar ratio in 0.1 M sodium bicarbonate (pH 8.5). The modified antibodies were purified by gel filtration and added at the ratios indicated in Table 3 to 20 mL of GNPs dispersed in water for 2 h to obtain conjugates. In the next step, 20 μL of 1 mM PEG-SH was added to stabilize the nanoparticles. Finally, the conjugates were centrifuged for 15 min (see Table 3) and resuspended, as indicated in Section 2.6.

### 2.9. Preparation of Immunochromatographic Test Strips

Reagents were applied to membranes comprising the assay system with an IsoFlow automatic dispenser (Imagene Technology; Lebanon, NH, USA). To form the control zone (CZ), a 1.0 mg/mL solution of GAMI antibodies in PBS containing 0.25% BSA, 0.25% sucrose, and 0.1% sodium azide (all *w/v*) was used. For the test zone (TZ), 1.0 mg/mL solutions of anti-cTnI antibodies and clone IC19 in the same buffer were used; 2.0 μL of both of the above solutions were applied per 1 cm of the working nitrocellulose membrane width. Conjugates of C-GNPs or S-GNPs with anti-cTnI antibodies, clone IC4, were applied to glass fiber PT-R7 membranes at dilutions corresponding to optical density 5.0 at 520 nm (16.0 μL per 1 cm of membrane width). The membranes with deposited immunoreagents were air-dried at room temperature for at least 20 h. These two membranes as well as the membrane for sample separation and the final adsorbing membrane were combined to assemble multimembrane composites, from which 3.5-mm-wide test strips were obtained using an automatic guillotine Index Cutter-1 (A-Point Technologies; Gibbstown, NJ, USA).

### 2.10. Immunochromatographic Assay and Data Processing

The assay was done at room temperature. The lower end of a test strip was dipped into an aliquot of the sample (70 μL) for 1 min and then placed on a horizontal surface. The result was checked within 10 min after sample application. The assaying of each sample was carried out in triplicate.

Digital images of the test strips were obtained with a Canon CanoScan 9000F scanner and analyzed with TotalLab software (Cleaver Scientific; Rugby, UK), as described in our previous paper [50]. The dependence of the intensity of TZ staining on the antigen concentration in the sample was processed using Origin 9.1 software (OriginLab Corp.; Northampton, MA, USA). The choice of TZ staining intensity as the plotted parameter instead of the often-considered TZ ratio and CZ intensities was based on the necessity of considering LFIA properties over the course of the tests’ storage. In addition to the test and control zones, different reactions took place and the TZ/CZ ratio changed significantly due to decreased binding in the control zone. TZ intensity, meanwhile, was more stable.

## 3. Results

### 3.1. Size and Shape Characterization of the Synthesized GNPs

**Figure 1:**
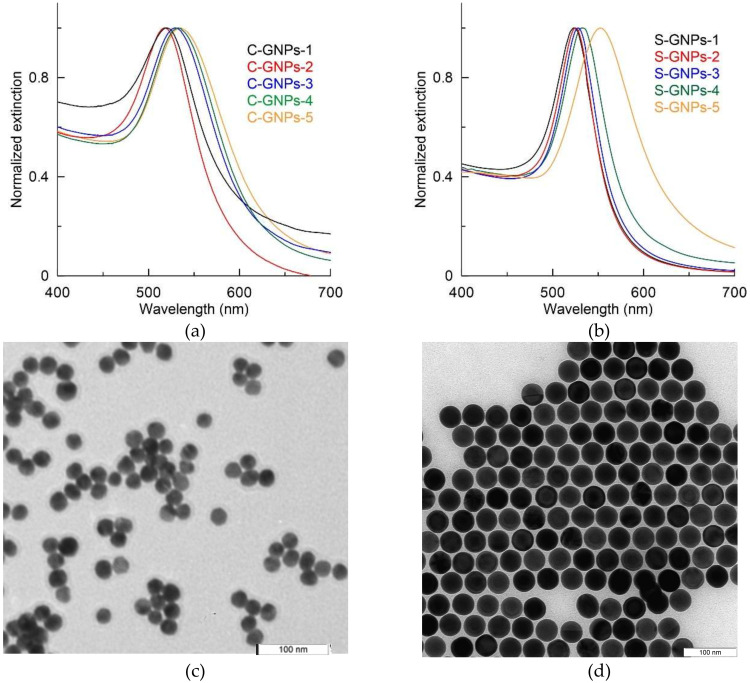
(**a**) Extinction spectra of citrate-capped quasispherical particles C-GNPs-1–C-GNPs-5. (**b**) Extinction spectra of superspherical particles S-GNPs-1–S-GNPs-5. (**c**) Representative TEM image of C-GNP-4. (**d**) Representative TEM image of S-GNP-3.

C-GNP and S-GNP series were synthesized with varied ratios of reactants (see Table 1 and Table 2) to reach different average nanoparticle diameters. In total, five C-GNPs and five S-GNP colloids were obtained. All of them were stable colloidal suspensions of red color, which is typical for nanodispersed gold. Figure 1a,b shows the colloid extinction spectra. For C-GNP, the position of the maximum extinction spectra depends on the amount of sodium citrate added during synthesis. It reached 518 nm for C-GNP-1, 520 nm for C-GNP-2, 527 nm for C-GNP-3, 532 nm for C-GNP-4, and 536 nm for C-GNP-5. The shift in the plasmonic peak to a longer wavelength range is indicative of a progressive increase in nanoparticle size. In the case of S-GNPs, extinction maximums reached values ranging from 522 to 563 nm, depending on the nanoparticle size. Note that the plasmonic peak of S-GNP had a lower width compared to C-GNP, which is indicative of higher sphericity and narrow size distribution. This observation was confirmed using TEM measurement (Figure 1c,d and Supporting Information, Figures S1 and S2).

For further characterization of the obtained preparations, GNP size and shape were analyzed using TEM. Table 4 summarizes microphotograph processing data. As can be seen, the variation in size was significantly lower for the S-GNP preparations, reaching 1.2–3.0%, in comparison with 7.0% or more for the commonly used C-GNPs. Both rows encompass the diameter range of 30–40 nm that is typically recommended for LFIA. However, the protocol for obtaining S-GNPs provides the possibility of extending the particle diameters to 90 nm, whereas C-GNPs of such size are known to be unstable. Besides, S-GNPs are characterized by a unified spherical shape, with minimal variation in the ellipticity index (see Table 4). Thus, the chosen approach leads to essential unification for geometrical parameters of the obtained GNPs. Images of the C-GNPs and S-GNPs are given in the Supporting Information, Figures S1 and S2.

### 3.2. Immunochromatographic Assay and Data Processing

The next stage of the study was the immobilization of antibodies on the surface of the obtained GNPs. The choice of antibody: GNP ratio for immobilization was based on the calculation of the necessary quantity of immunoglobulin molecules to reach a monolayer coverage of the GNPs’ surface. Table 5 gives the corresponding calculations. The surface necessary to immobilize one immunoglobulin G molecule was estimated as 25 nm^2^.

### 3.3. Immunochromatographic Assay and Data Processing

**Figure 2:**
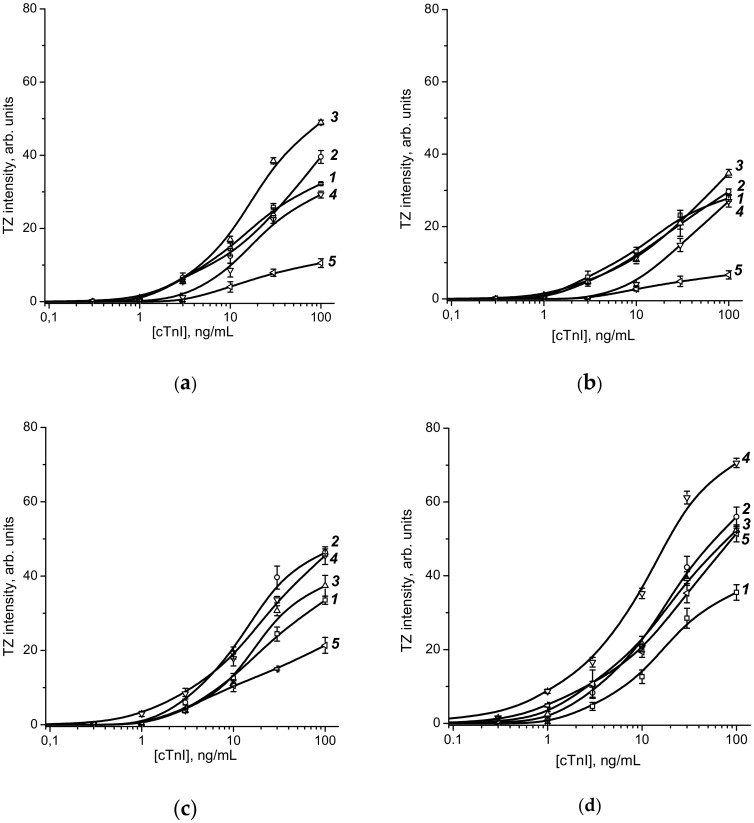
Dependencies of the intensities of staining of the test zone on the concentration of antigens in the sample for conjugates C-GNPs-1–C-GNPs-5 (**a**,**b**) and S-GNPs-1–S-GNPs-5 (**c**,**d**) during adsorption (**a**,**c**) and covalent (**b**,**d**) immobilization of antibodies. All measurements were performed in triplicate.

To choose the optimal conditions for LFIA, the obtained conjugates with adsorbed and cross-linked antibodies were used as labels, and the intensity of the test zone was measured as a function of the cTnI concentration. The obtained data are shown in Figure 2.

Based on the obtained concentration dependencies, the LoD values were determined for all four series of the conjugated GNPs, namely adsorption and covalent conjugates of C–GNPs and S-GNPs. These values are summarized in Table 6. To estimate the efficiency of visual assessment of the assay results based on the intensity of TZ coloration, the maximal saturating levels of these colorations for all tested kinds of GNP–antibody conjugates are summarized in Table 7.

Table 6 demonstrates that the S-GNPs-4 with covalent immobilization is the best choice to reach a lower sensitivity. When comparing 10 pairs with adsorption and covalent immobilization (Table 6), we can see that the difference between them is typically no more than twofold, and there are variants of a smaller LOD for both covalent and adsorption immobilization. The factors affecting these differences include the risks of antibody desorption (worsening parameters for adsorption immobilization) and a decrease in the surface density of active antibody molecules due to modification inactivation or non-oriented fixation (worsening parameters for covalent immobilization). Apparently, a final comparison of the two options for immobilization is possible only for a significantly wider range of drugs, including antibodies to different antigens.

As can be seen in the case of using C-GNPs as labels, the most effective binding of conjugates in the test zone was obtained for C-GNP3 particles with an average size of 33.7 nm. Note that the adsorption immobilization of antibodies was more effective than the covalent binding. In contrast, S-GNP covalent immobilization of antibodies gives a more intense signal than adsorption. The optimal S-GNP size was 64.5 nm. The chosen variants are marked in red in Table 6 and Table 7.

**Figure 3:**
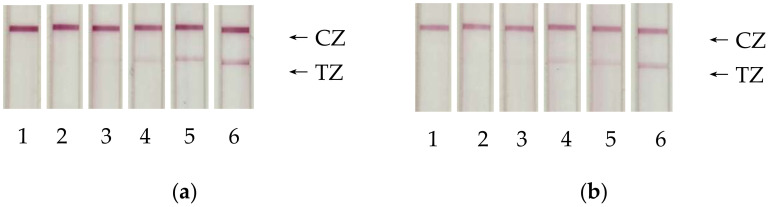

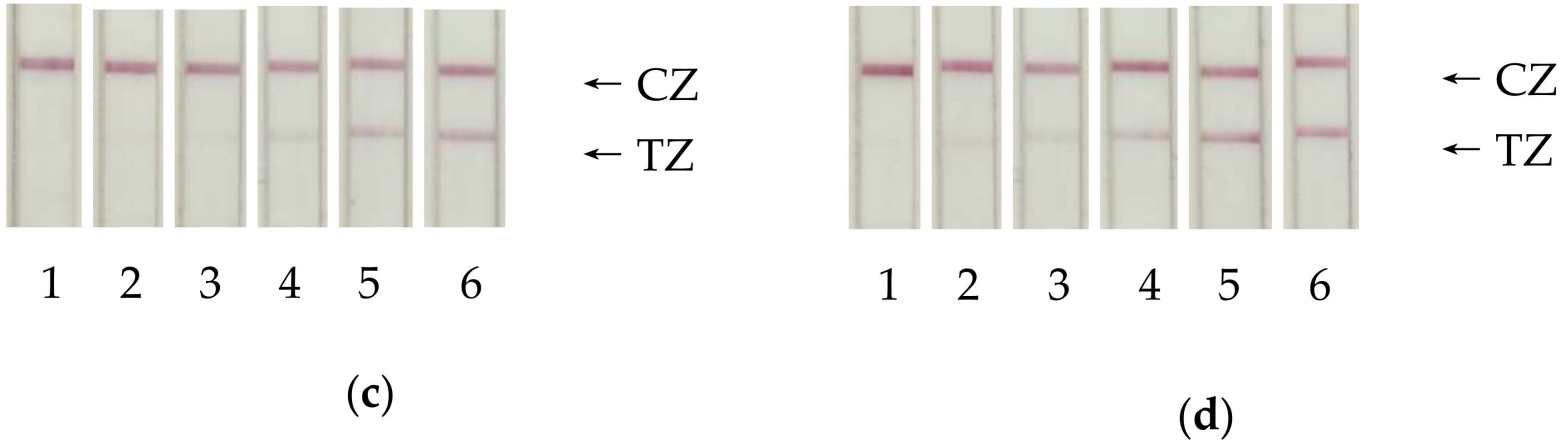
Images of test strips after immunochromatographic detection of cTnI at concentrations of 0.3 (1), 1 (2), 3 (3), 10 (4), 30 (5), and 100 (6) ng/mL. Panels (**a**,**b**) correspond to the conjugate C-GNPs-3 (33.7 nm)–IC4; (**c**,**d**) correspond to the conjugate S-GNPs-4 (64.5 nm)–IC4. Panels (**a**,**c**) correspond to adsorption immobilization of antibodies, (**b**,**d**) to covalent immobilization of antibodies. CZ: control zone, TZ: test zone.

Based on this choice, we further measured the LoD for LFIA strips with optimal GNP sizes, namely for conjugates C-GNPs-3–IC4 (average diameter of C-GNPs, 33.7 nm) and S-GNPs-4–IC4 (average diameter of S-GNPs, 64.5 nm) for both immobilization regimes. The images of test-strips for the assays of various cTnI concentrations are shown in Figure 3.

**Figure 4:**
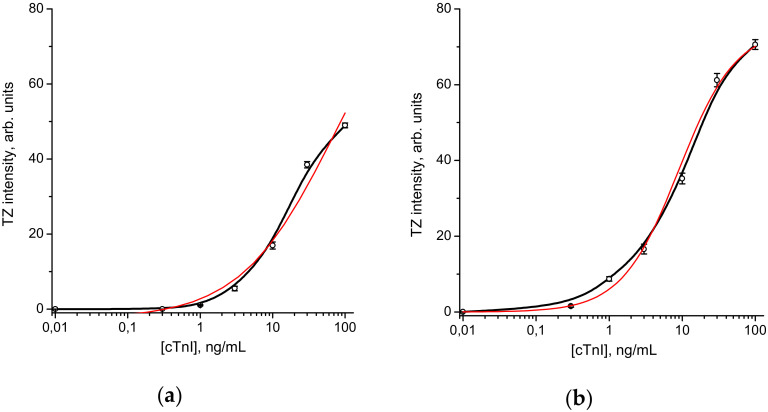
Dependence of the intensity of TZ staining on the concentration of cTnI in the sample was approximated (red line) by the function: *y* = (*A* − *D*)/(1 + (*x*/*C*)*^B^*) + *D*. For the conjugate C-GNPs-3 (33.7 nm)–IC4 with adsorption immobilization of antibodies (**a**) A = 0.07, D = 49.15, C = 23.0, and B = 2.62; for the conjugate sGNPs-4 (64.5 nm)–IC4 with covalent immobilization of antibodies (**b**), A = 0.04, D = 75.0, C = 8.98, and B = 1.11. All measurements were performed in triplicate.

The best variants for both GNPs demonstrate a significant increase in sensitivity (Figure 4). The possible S-GNP detection limit was 1.2 ng/mL. For common C-GNPs, the corresponding value was 9.9 ng/mL. Thus, the proposed new immunochromatographic label provided an 8-fold improvement in assay sensitivity.

This difference is caused by a combination of two factors: higher intensity of coloration for the same cTnI content and lower signal variation for the S-GNPs-based assays, providing reliable distinguishing of positive and negative samples for low levels of coloration. According to the intensity of the test zone coloration, the s-GNPs are twice as bright as c-GNPs (at 100 ng/mL, the corresponding values are 75 and 49 arb. units, at 10 ng/mL, −35 and 17 arb. units).

The presented data differ with the earlier published [37] comparison of GNPs with Au cores and small CTAC shells that cover the diameter range from 16 to 115 nm with high homogeneity (RSD 2−3%). In the previous study, the monotonous dependence of LoD on the GNP diameter was found to reach 0.157 pg/mL of cTnI for the largest GNPs. However, this work did not considered long-time stability for supersspherical GNPs. Our more detailed studies indicated that the highly spherical GNPs with maximal diameters (>65 nm) demonstrated a limited colloidal stability after conjugation with antibodies. It caused non-specific binding of the conjugates with the working membrane in the course of LFIA and lost sensitivity.

The freshly prepared S-GNPs and C-GNPs demonstrated good colloidal stability with reproducible adsorption spectra and the absence of visible precipitates, independent of their size. The DLS data indicated the occasional presence of a small (0.1–0.5%) quantity of aggregates with diameters in the range of 100 nm–1 mkm (depending on the initial size of particles). These affects were not in strong accordance with GNP type and did not lead to further increased aggregation (see Figure S3 in the Supporting Information). More pronounced and reproducible regularities were found after long-term storage of the GNP preparations conjugated with antibodies. The stability of colloidal solutions for C-GNPs and their conjugated derivatives depended significantly on their size. Visible precipitates occurred for the average diameter of C-GNPs, which was equal to 47.5 after one to two months of storage (see Figure S4 in the Supporting Information). This effect may create worse sensitivity in the assay with these GNPs as a label. This finding is in accordance with earlier presented data about C-GNPs for large diameters that needed additional surface modifications to provide stability [38]. The S-GNPs conjugated with antibodies possess long-time stability of colloidal solutions based on spectral and DLS data in a range of diameters up to 64.5 nm. Two tested S-GNP preparations with large diameters (i.e., 90.4 and 115.3 nm) demonstrated shifts in DLS spectra after two months of storage (see Figure S5 in the Supporting Information). Due to this, the advantages of S-GNPs can be successfully transformed to lower LODs only in a range of up to 64.5 nm, as stated above.

Physical antibody mechanisms bound to a GNP surface for different preparations need additional investigation using sophisticated equipment to estimate the orientation of immobilized antibodies, their conformational rearrangement, storage of antigen-binding ability, kinetic binding parameters, and steric hindrance for interaction with globular antigen molecules. The existing concepts consider surface defects at the atomic level and changing curvature as factors influencing possible partial inactivation of immobilized antibodies, but this interconnection has yet to be grounded as a priority and universal factor. In any case, two reasons should be taken into consideration when evaluating identified S-GNP benefits. The first is minimal differences in the properties of the S-GNPs’ surface, providing the possibility to maximize the cases of efficient antibody binding under the chosen optimal reaction media. The second is the use of additional stabilizers in the course of the two-step S-GNP synthesis. Due to this, the effect of colloidal instability is moved for S-GNPs to higher diameters, as compared with C-GNPs, and the intensity of coloration for labeling individual analyte molecules increased in accordance with the growth in the label’s size. Note that both effects are analyte-independent, which determines their high potential for LFIAs of other compounds.

The presented study gives an experimental estimation of LODs that integrates both basic properties of the conjugates (that may be characterized in terms of optical density, composition, etc.) and specific properties connected with aggregation stability, steric availability of a particular antigen for binding to immobilized antibodies, and so on. The compositions of all these factors show the need for more experimental studies. The identified regularities are impossible for prognostic assessment of the reactivity of conjugates and LODs achieved with their help.

## 4. Conclusions

The presented study demonstrated a significant improvement in lateral flow immunoassay sensitivity by using superspherical gold nanoparticles instead of the commonly used quasispherical citrate-capped gold nanoparticles via the Turkevich–Frens technique. The known modifications of C-GNPs synthesis do not give such monodispersity as the super-spherical preparation under consideration in this paper. The proposed superspherical GNPs have advantages in unified size and shape that are unattainable for alternative preparations. Therefore, these GNPs were compared with the common Turkevich–Frens C-GNPs. They caused a big gain in sensitivity in the immunochromatographic analysis.

This improvement is the result of two factors: (i) more efficient immobilization of antibodies on the nanoparticle surface without variations in curvature and (ii) the possibility of using larger nanoparticles with increased quantity of binding sites of immobilized antibodies. The resulting lowering of the detection limit for troponin I gives reason for further extension of the field of application to other practically important analytes.

## Figures and Tables

**Table 1:** Proportions of reagents for gold nanoparticles preparation using the Frens method.

Sample	Volume of Mixed Reactants, mL		
	1% HAuCl_4_	Water	1% Sodium Citrate
C-GNPs-1	1.0	94.0	5.0
C-GNPs-2	1.0	96.0	3.0
C-GNPs-3	1.0	97.5	1.5
C-GNPs-4	1.0	98.0	1.0
C-GNPs-5	1.0	98.25	0.75

**Table 2:** Proportions of reactants for the second step of the spherical gold nanoparticle preparation.

Sample	Volume of Mixed Reactants, mL			
	0.1 M CTAC	10 mM AA	10-nm GNP	2 mM HAuCl_4_
S-GNPs-1	40	2.6	2	10
S-GNPs-2	40	2.6	0.5	10
S-GNPs-3	40	2.6	0.15	10
S-GNPs-4	40	2.6	0.1	10
S-GNPs-5	40	2.6	0.05	10

**Table 3:** Regimes for the separation of GNP-antibody conjugates.

Conjugate	Acceleration, g	Conjugate	Acceleration, g
C-GNPs-1–IC4	27,000	S-GNPs-1–IC4	25,000
C-GNPs-2–IC4	25,000	S-GNPs-2–IC4	15,000
C-GNPs-3–IC4	15,000	S-GNPs-3–IC4	10,000
C-GNPs-4–IC4	12,000	S-GNPs-4–IC4	8000
C-GNPs-5–IC4	10,000	S-GNPs-5–IC4	5000

**Table 4:** Geometrical parameters of the obtained series for C-GNPs and S-GNPs.

Sample	Average Diameter, nm	Standard Deviation, %	Ellipticity Index	Number of Particles
C-GNPs-1	18.6	12.4	1.17 ± 0.19	305
C-GNPs-2	21.5	7.0	1.23 ± 0.20	353
C-GNPs-3	33.7	8.9	1.17 ± 0.35	283
C-GNPs-4	39.5	12.7	1.30 ± 0.30	325
C-GNPs-5	47.5	9.9	1.28 ± 0.19	278
S-GNPs-1	20.2	3.0	1.01 ± 0.01	521
S-GNPs-2	36.2	1.7	1.01 ± 0.01	554
S-GNPs-3	48.7	2.1	1.02 ± 0.02	547
S-GNPs-4	64.5	1.2	1.03 ± 0.02	548
S-GNPs-5	90.4	1.7	1.02 ± 0.02	606

**Table 5:** The choice of the antibody: GNP ratio for their conjugation.

Sample	Average Diameter, nm	GNPs’ Surface Area, nm^2^	Antibody: GNP Ratio for Immobilization	IgG Concentration, µg/mL
C-GNPs-1	18.6	1086	43	7.02
C-GNPs-2	21.5	1452	58	5.9
C-GNPs-3	33.7	3566	143	4.55
C-GNPs-4	39.5	4900	196	3.34
C-GNPs-5	47.5	7085	283	2.76
S-GNPs-1	20.2	1256	50	15.24
S-GNPs-2	36.2	4069	163	8.04
S-GNPs-3	48.7	7539	302	5.72
S-GNPs-4	64.5	13,267	531	6.44
S-GNPs-5	90.4	26,002	1040	3.0

**Table 6:** LoDs of cTnI detection for LFIAs with different antibody–GNP conjugates.

Conjugation Approach	LoDs of cTnI LFIA, ng/mL, for Various GNP Preparations				
	**C-GNPs-1 (18.6 nm)**	**C-GNPs-2 (21.5 nm)**	**C-GNPs-3 (33.7 nm)**	**C-GNPs-4 (39.5 nm)**	**C-GNPs-5 (47.5 nm)**
Adsorption	13.2 ± 0.15	10.1± 0.55	9.9 ± 0.24 *	15.4 ± 0.43	35.4 ± 0.65
Covalent	13.4 ± 0.60	12.5 ± 0.45	12.7 ± 0.14	20.3 ± 0.35	>100
	**S-GNPs-1 (20.2 nm)**	**S-GNPs-2 (36.2 nm)**	**S-GNPs-3 (48.7 nm)**	**S-GNPs-4 (64.5 nm)**	**S-GNPs-5 (90.4 nm)**
Adsorption	2.9 ± 0.13	3.3 ± 0.31	2.7 ± 0.20	2.0 ± 0.05	4.7 ± 0.25
Covalent	3.4 ± 0.14	2.8 ± 0.25	1.5 ± 0.15	1.2 ± 0.08 *	3.8 ± 0.15

* The chosen variants of antibody–GNP conjugates are marked in red.

**Table 7:** Maximal coloration * of test zone for LFIAs of cTnI with different antibody–GNP conjugates.

Conjugation Approach	Maximal Coloration of Test Zone or cTnI LFIA, Arb. Units, for Various GNP Preparations				
	**C-GNPs-1 (18.6 nm)**	**C-GNPs-2 (21.5 nm)**	**C-GNPs-3** **(33.7 nm)**	**C-GNPs-4 (39.5 nm)**	**C-GNPs-5 (47.5 nm)**
Adsorption	32.2 ± 0.30	39.55 ± 1.75	49.0 ± 0.64 **	29.25 ± 0.95	10.5 ± 1.2
Covalent	27.85 ± 0.85	29.65 ± 0.75	34.7 ± 1.1	27.0 ± 1.6	6.65 ± 1.15
	**S-GNPs-1 (20.2 nm)**	**S-GNPs-2 (36.2 nm)**	**S-GNPs-3** **(48.7 nm)**	**S-GNPs-4 (64.5 nm)**	**S-GNPs-5 (90.4 nm)**
Adsorption	33.5 ± 1.1	46.35 ± 0.95	37.4 ± 2.8	45.5 ± 2.4	21.35 ± 2.15
Covalent	35.5 ± 2.1	56.0 ± 2.6	52.35 ± 0.35	70.6 ± 1.3 **	51.5 ± 2.3

* The presented values accord to the cTnI concentration equal to 100 ng/mL, which typically accords to saturation of binding sites in the control zone of the test strip. ** The chosen variants of antibody–GNP conjugates are marked in red.

## Supplementary Materials

The following are available online at https://www.mdpi.com/1424-8220/20/12/3608/s1. Figure S1: TEM images of C-GNPs, preparations 1–5; Figure S2: TEM images of S-GNPs, preparations 1–5; Figure S3: Examples of DLS measurements for C-GNPs (a, diameter 18.6 nm) and S-GNPs (b, diameter 20.2 nm) preparations; Figure S4: Diameters distributions of C-GNPs-antibodies conjugates (data of DLS measurements) for with an average diameters of 21.5 (a), 33.7 (b), 39.5 (c) and 47.5 (d) nm. The black curves correspond to the freshly prepared conjugates; the red curves were obtained after 2 months of storage at 4 °C; Figure S5 Diameters distributions of S-GNPs-antibodies conjugates (data of DLS measurements) for with an average diameters of 48.7 (a), 64.5 (b), 90.4 (c) and 115.3 (d) nm. The black curves correspond to the freshly prepared conjugates; the red curves were obtained after 2 months of storage at 4 °C.
