# Supplementary material for: Advantages of Highly Spherical Gold Nanoparticles as Labels for Lateral Flow Immunoassay
Source: Sensors (Basel). 2020 Jun 26;20(12):3608. doi: 10.3390/s20123608 (PMC7348961; doi:10.3390/s20123608)
Supplement: Supplementary file 1 [file sensors-20-03608-s001.pdf]

sensors

Supplementary Materials

Advantages of highly spherical gold nanoparticles as labels for lateral flow immunoassay

Nadezhda A. Byzova,[1] Anatoly V. Zherdev,[1] Boris N. Khlebtsov,[2,*] Andrey M. Burov,[2] Nikolai G. Khlebtsov,[2,3] Boris B. Dzantiev[1,*]

[1] *A.N. Bach Institute of Biochemistry, Research Center of Biotechnology, Russian Academy of Sciences, 119071 Moscow, Russia*
[2] *Institute of Biochemistry and Physiology of Plants and Microorganisms, Russian Academy of Sciences, 410049 Saratov, Russia*
[3] *Saratov State University, 410012 Saratov, Russia*

* Correspondence: dzantiev@inbi.ras.ru; Tel.: +7-495-954-3142 khlebtsov_b@ibppm.ru ; Tel.: +7-8452-97-0403

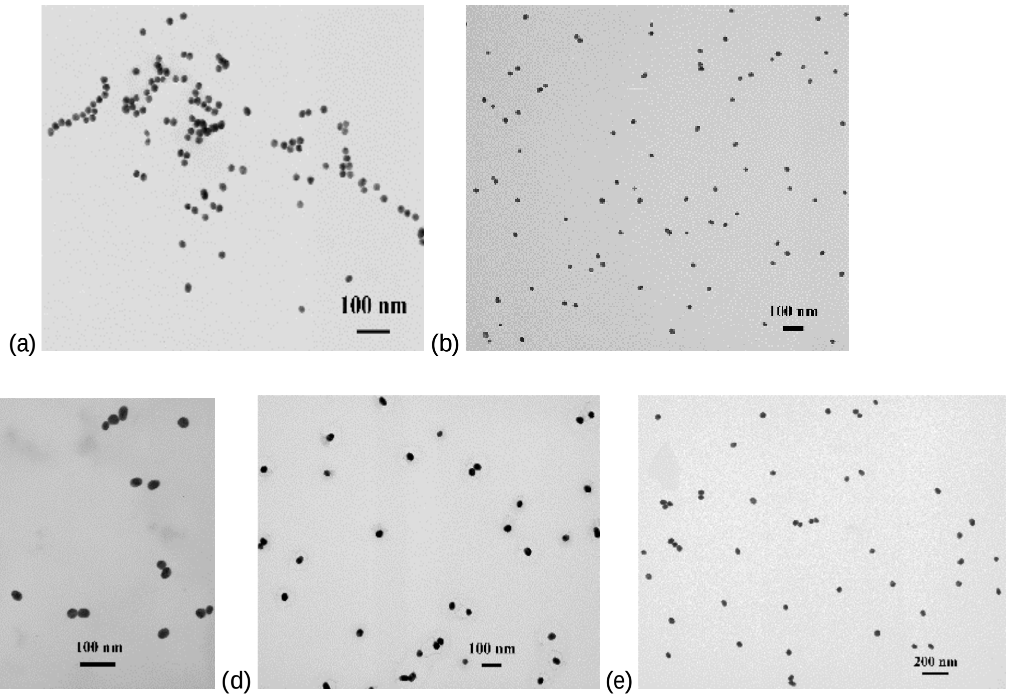

Fig. S1. TEM images of C-GNPs, preparations 1–5 (a–e, respectively).

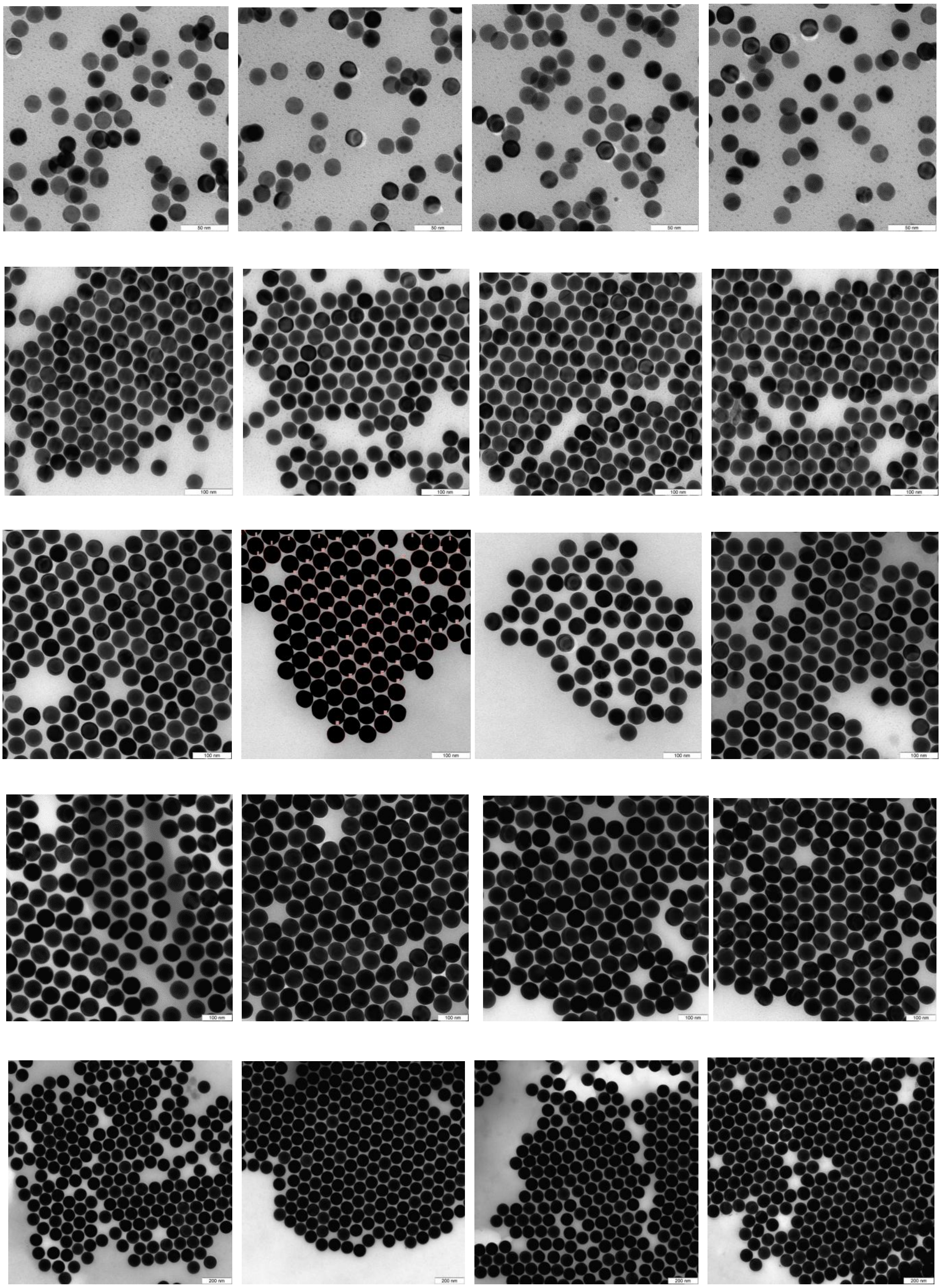

(a)

(b)

(c)

(d)

(e)

Fig. S2. TEM images of S-GNPs, preparations 1–5 (a–e, respectively).

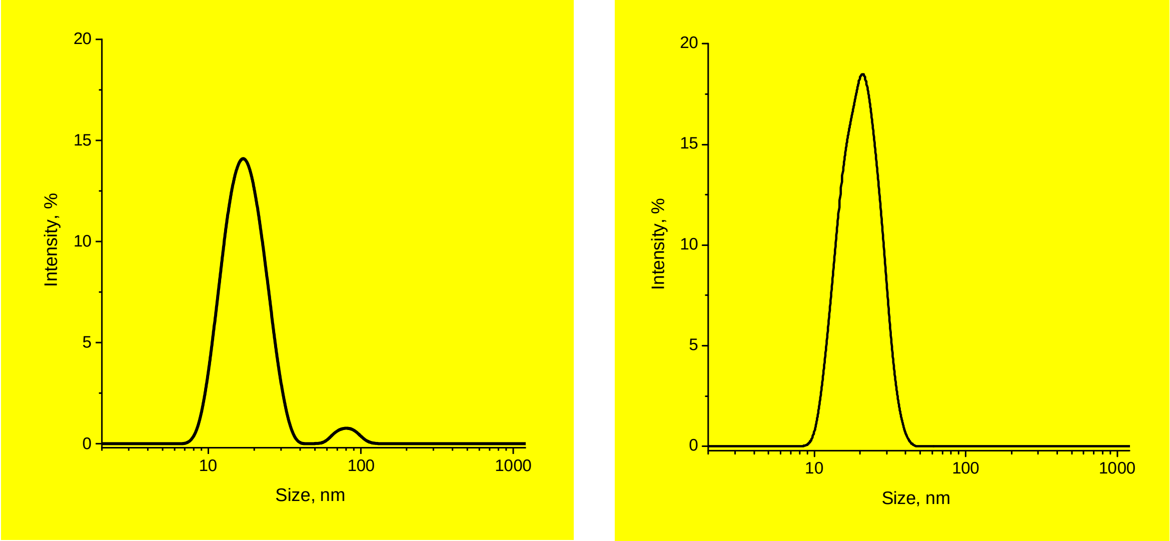

a b

Fig. S3. Examples of DLS measurements for C-GNPs (a, diameter 18.6 nm) and S-GNPs (b, diameter 20.2 nm) preparations

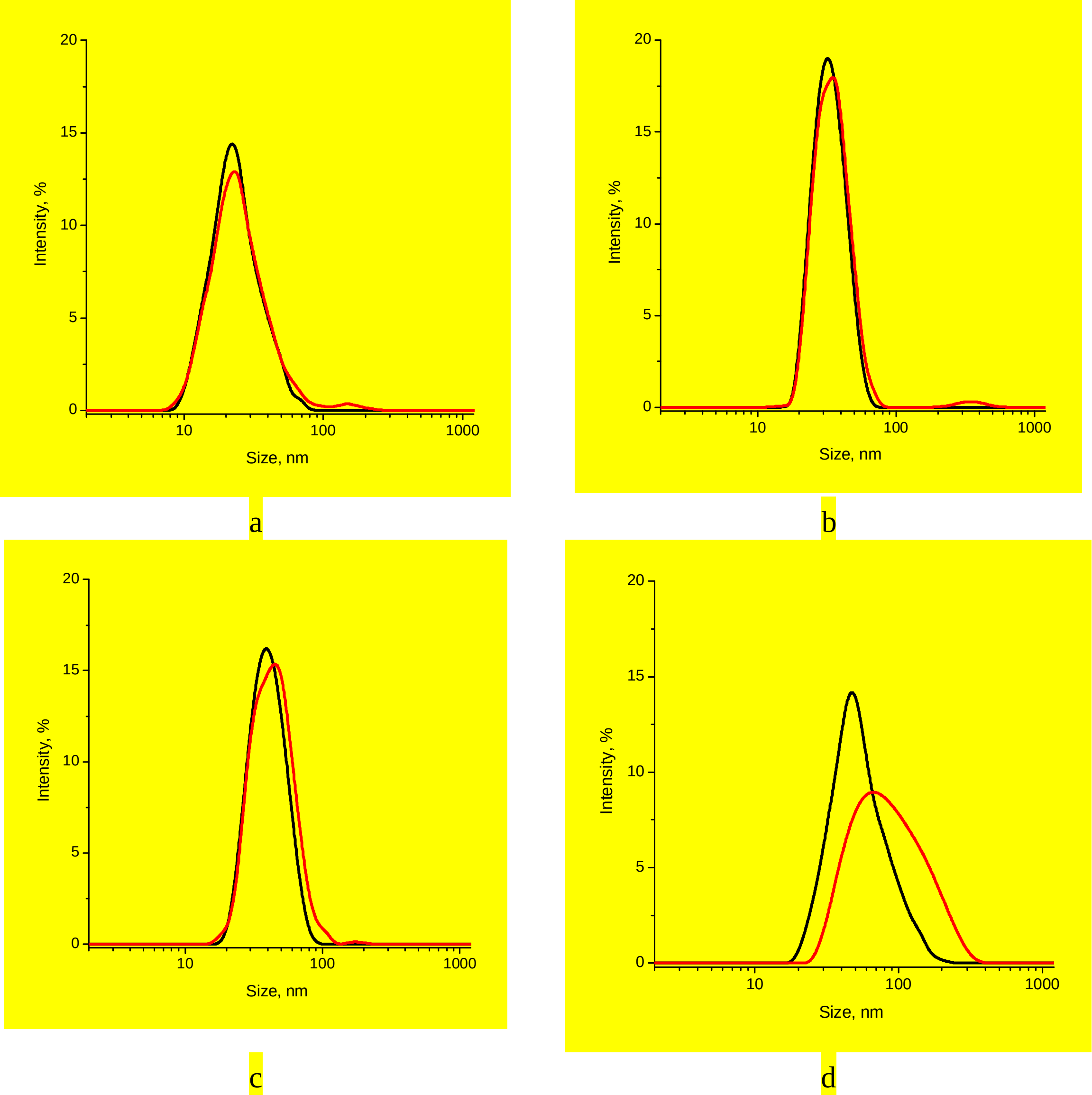

Fig. S4. Diameters distributions of C-GNPs-antibodies conjugates (data of DLS measurements) for with an average diameters of 21.5 (a), 33.7 (b), 39.5 (c) and 47.5 (d) nm. The black curves correspond to the freshly prepared conjugates; the red curves were obtained after 2 months of storage at 4 °C.

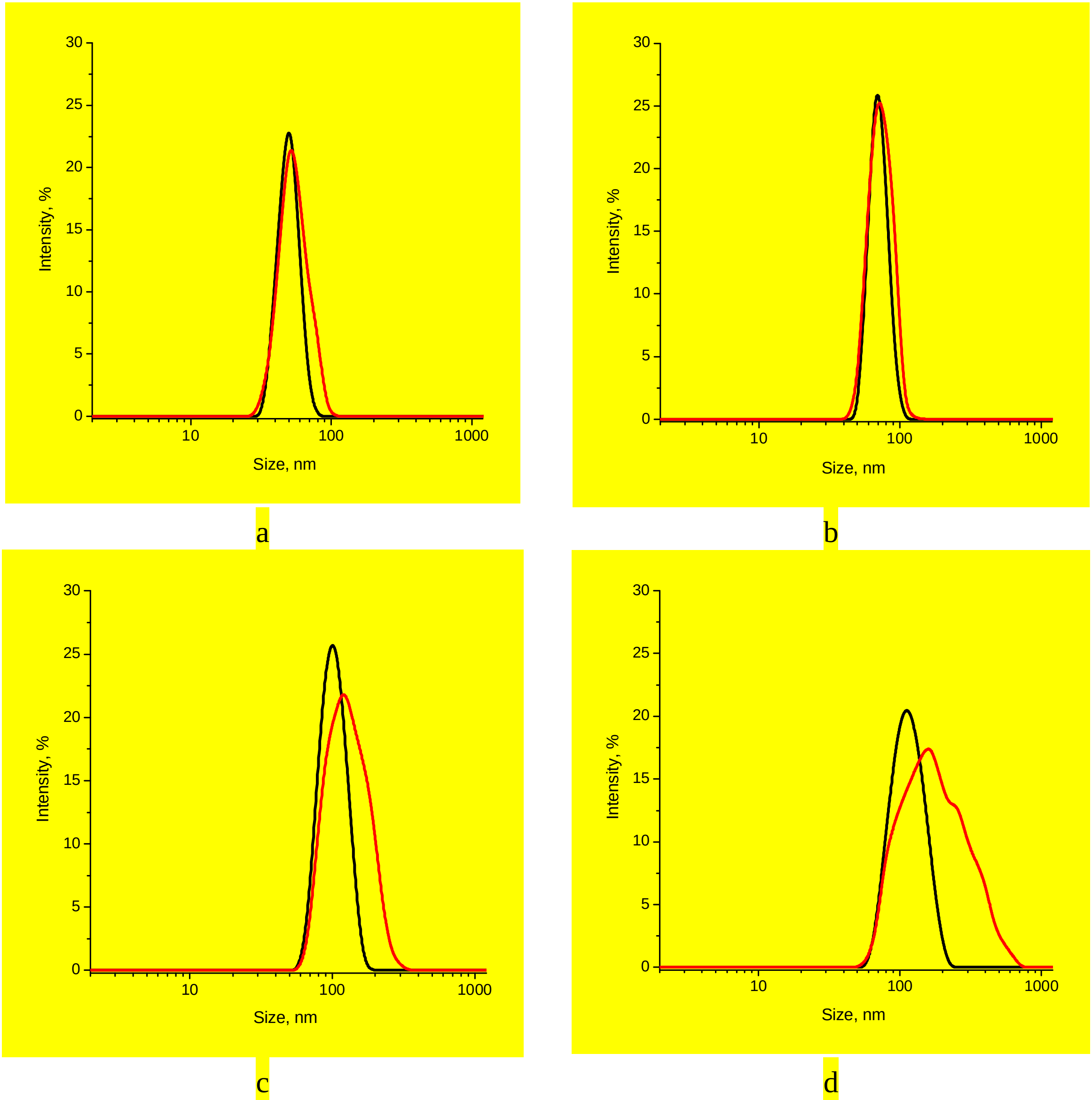

Fig. S5. Diameters distributions of S-GNPs-antibodies conjugates (data of DLS measurements) for with an average diameters of 48.7 (a), 64.5 (b), 90.4 (c) and 115.3 (d) nm. The black curves correspond to the freshly prepared conjugates; the red curves were obtained after 2 months of storage at 4 °C.
